# Chitosan Inhibits *Helicobacter pylori* Growth and Urease Production and Prevents Its Infection of Human Gastric Carcinoma Cells

**DOI:** 10.3390/md18110542

**Published:** 2020-10-29

**Authors:** Shun-Hsien Chang, Pei-Ling Hsieh, Guo-Jane Tsai

**Affiliations:** 1 Institute of Food Safety and Risk Management, National Taiwan Ocean University, Keelung 20224, Taiwan; lewis@mail.ntou.edu.tw; 2 Department of Food Science, National Taiwan Ocean University, Keelung 20224, Taiwan; peiling615@yahoo.com.tw; 3 Center for Marine Bioenvironment and Biotechnology, National Taiwan Ocean University, Keelung 20224, Taiwan

**Keywords:** chitosan, antibacterial activity, *Helicobacter pylori*, urease activity, TSGH9201

## Abstract

This study investigated the effects of shrimp chitosan with 95% degree of deacetylation (DD95) in combination with clinical antibiotics on the growth and urease production of *Helicobacter pylori*. The inhibitory effect of DD95 on the adherence of *H. pylori* to the human intestinal carcinoma cells (TSGH9201) was also investigated. Five strains of *H. pylori*, including three standard strains and two strains of clinical isolates were used as the test strains. The inhibitory effects of DD95 on growth and urease production of various strains of *H. pylori* increased with increasing DD95 concentration and decreasing pH values from pH 6.0 to pH 2.0. Urease activity of *H. pylori* at pH 2.0 in the presence of 4000 μg/mL of DD95 decreased by 37.86% to 46.53%. In the presence of 50 μg/mL antibiotics of amoxicillin, tetracycline, or metronidazole at pH 6.0 and pH 2.0, *H. pylori* counts were decreased by 1.51–3.19, and 1.47–2.82 Log CFU/mL, respectively. Following the addition of 4000 μg/mL DD95 into the 50 μg/mL antibiotic-containing culture medium with pH 6.0 and pH 2.0, overall *H. pylori* counts were strongly decreased by 3.67–7.61 and 6.61–6.70 Log CFU/mL, respectively. Further, DD95 could inhibit the adherence of *H. pylori* on TSGH 9201 cells, as evidenced by fluorescent microscopy and thus may potentially protect against *H. pylori* infection.

## 1. Introduction

Epidemiological studies have found that *Helicobacter pylori* is closely associated with human gastrointestinal diseases, including chronic gastritis, gastric ulcer, duodenal ulcer, atrophic gastritis, mucosa-associated lymphoid tissue lymphoma, gastric cancer, and adenocarcinoma [1]. Over 50% of population was infected by *H. pylori* [2] and approximately 6.2% of cancer cases reported worldwide is associated with *H. pylori* infection [3]. Treatment strategies using a single antibiotic plus a proton pump inhibitor (dual therapy) or two antibiotics plus a proton pump inhibitor or bismuth (triple therapy) have been adopted to eradicate *H. pylori.* The eradication rate for triple therapy using a proton pump inhibitor (operazole, lansoprazole) plus two antibiotics (e.g., amoxicillin [4], levofloxacin, metronidazole [5], or tetracycline [6]) is between 85 and 95%. However, recently the eradication treatment efficacy of *H. pylori* has worsened [7]. The drawbacks result from a significant amount of side effects, low patient compliance, and drug resistance, or poor antibacterial effects of the antibiotic in a low pH environment and thus, this approach frequently results in treatment failure [8]. In particular, the therapeutic approach is not always completely curative; thus, alternative therapeutic agents are needed [8,9,10].

Chitosan, a partially deacetylated chitin (poly-β-(1→4)*N*-acetyl-d-glucosamine) is a natural product found in shrimp, crab shells, and fungal cell wall. This polysaccharide and its derivatives have attracted attention as biomedical materials because of their good biodegradability [11], bioadhesion [12], and biocompatibility [13] properties with no observable immunogenicity, toxicity or side effects [14]. Given its excellent antibacterial activity, chitosan has been widely used for food preservation [15]. Several studies have shown that chitosan’s molecular weight (MW) is a crucial factor of its antimicrobial properties. Our previous report concluded that the correlation between chitosan MW and its antibacterial properties was dependent on the pH value of the reaction mixture [16].

In this study, we prepared shrimp chitosan with 95% deacetylation (DD95) and demonstrated that this preparation exerts strong antibacterial activity against *H. pylori* and effectively reduces urease activity of test strains. Combination of inhibitory effects of DD95 with amoxicillin, tetracycline, or metronidazole antibiotics on *H. pylori* strains were also observed. Furthermore, DD95 can reduce the adhesion of *H. pylori* to human gastric cancer cells (TSGH 9201) as observed using fluorescence microscopy. 

## 2. Results and Discussion

### 2.1. Effects of DD95 Chitosan on Growth and Urease Production of H. pylori

**Figure 1:**
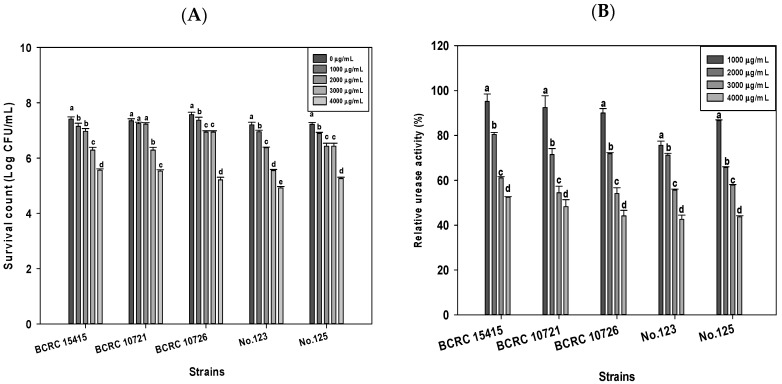
Survival counts, (**A**) and relative urease production of, (**B**) 5 strains of *H. pylori* in serum-added BHIB containing various concentrations of DD95 at 37 °C for 24 h. a–e, Different letters in the bar for the same test item are significantly different (*p* < 0.05).

Based on the method described by Chang et al. [16], a chitosan with a MW of 220 kDa and 95% degree of deacetylation (DD95) was chemically prepared from shrimp chitin. Five strains of *H. pylori*, including 3 standard strains (BCRC 15415, BCRC 10721, BCRC 10726) and 2 clinical isolates (No. 123, and 125) were used as the test strains. The antibacterial effects induced by DD95 against these test strains in serum added brain heart infusion broth (sBHIB) with various concentrations of DD95 in micro-aerobic culture conditions at 37 °C for 1 day are shown in Figure 1A. A dose-dependent reduction of *H. pylori* counts induced by DD95 was observed. The survival counts ranging from 4.92 to 5.56 Log CFU/mL for the tested strains at 4000 μg/mL DD95 were obtained, which were significantly lower than those (7.20 to 7.57 Log CFU/mL) of the controls for each strain. All the 5 tested strains showed similar susceptibility to DD95.

Effects of DD95 on the relative urease activity of *H. pylori* at 37 °C for 24 h are shown in Figure 1B. The relative urease activity of each strain tested significantly decreased with increasing concentrations of DD95. The relative urease activities ranged from 42.58% to 52.40% for the 5 tested strains at 4000 ppm DD95. The production of bacterial urease leads to the production of ammonia, which maintains the pH of the periplasm and cytoplasm of bacteria close to neutral even in the case of gastric acid shock, allowing the bacteria to survive and colonize the stomach mucosa [17].Therefore, a decrease in urease activity observed across *H. pylori* standard strains and clinical isolates in study is of therapeutic significance [14,18].

The *H. pylori* strains BCRC 10726, No. 123, and 125 were chosen to further investigate the effects of pH on bacterial growth and urease production in sBHIB medium with or without 4000 DD95 chitosan. In the presence of 4000 μg/mL DD95, the bacterial survival count decreased with decreasing pH and the survival percentage ranged from 0.53% to 10.02% for the 3 tested strains at pH 2.0 to 6.0, when compared to the survivals of control strains. At pH 7 and pH 8, limited antibacterial effects by DD95 were observed and accordingly, much higher survival percentages ranging from 89.90% to 94.30% were achieved (Table 1). Furthermore, *H. pylori* cells are quite resistant to acidic conditions, thus the bacterial count of the control groups reduced only by 0.35 to 0.47 Log CFU/mL, compared to pH 7.0 and pH 3.0 conditions.

In the absence of DD95, the highest urease activities as revealed by the absorbance at 550 nm (OD_550_) in Table 2 for the 3 tested strains were obtained in sBHIB at pH 4.0. The increase or decrease of pH values from pH 4.0 resulted in the changes in urease activity, and especially at pH 7 and 8, urease activity decreased significantly. In the presence of 4000 μg/mL DD95, the relative urease activity for the 3 tested strains increased with increasing pH of the sBHIB culture. In particular, at pH 7 and 8, limited inhibitory effects induced by DD95 were observed. The antibacterial activity of chitosan relies on its positive charge [16,19], thus a greater positive charge of the chitosan molecule was obtained in more acidic conditions. Accordingly, the inhibitory effects of DD95 on bacterial growth and urease production by *H. pylori* increased with decreasing pH values from pH 6.0 to pH 2.0. In addition, the larger molecular size of DD95 did not exert any antibacterial activity at pH values of 7.0 or above [16], likewise, limited inhibition on bacterial growth and urease production was observed.

It is known that chitosan has good antibacterial activity against many food pathogens and spoilage bacteria. For example, the minimal lethal concentration (MLC) of the chitosan with 95% DD and 51 kDa against the Gram negative bacteria of *Escherichia coli*, *Pseudomonas. aeruginosa*, *Shigella. dysenteriae*, *Vibrio cholerae* and *V. parahaemolyticus*, and Gram positive bacteria of *Bacillus cereus*, *Listeria monocytogenes* and *Staphylococcus aureus* were in the range of 50–200 μg/mL [20]; while the MLC of the chitosan (95% DD, 220 KDa) against *Clostridium perfringens* was 250 μg/mL [21]. In this study, the chitosan at 4000 μg/mL, the concentration of which was much higher than the MLCs mentioned above, could not completely inhibit the growth of *H. pylori.* The antibacterial mechanism of chitosan is through binding the positively charged groups of chitosan with negatively charged groups on the bacterial surface due to electrostatic attraction, which in turn causes perforation or changes in cell membranes, and finally causes bacterial death [16]. The fact that *H. pylori* is much more resistant to chitosan, than the bacteria mentioned above, may be related to the differences in the surface structure between *H. pylori* and other bacteria. This merits further study in the future.

### 2.2. Combination of DD95 Chitosan and Antibiotics against H. pylori

Pathogenic microorganisms (including *H. pylori*) have developed drug resistance, thus antimicrobial chemotherapy often fails to achieve the expected success in eradicating microbial infections. Histological studies have shown that gastritis can be cured by eradicating *H. pylori* from the gastric mucosa [1]. The eradication of this pathogen not only heals ulcers, but also reduces the recurrence rate and is fundamentally curative for peptic ulcers [22]. Although, triple therapy, including amoxicillin plus other antibiotics is currently used, this strategy is complicated, has obvious side effects and compliance issues, and often leads to relapse [23]. Therefore, the impact of amoxicillin, tetracycline, and metronidazole plus 4000 μg/mL DD95 against *H. pylori* were evaluated in sBHIB at pH 6.0 and pH 2.0, as shown in Table 3 and Table 4, respectively.

As shown in Table 3, the survival counts for the 3 strains tested in sBHIB (pH 6.0) containing 4000 μg/mL DD95 ranged from 5.13 to 5.39 Log CFU/mL, which was about a 2 Log reduction compared to the survival of the control strains (7.20 to 7.61 Log CFU/mL). The three individual antibiotics tested showed much higher antibacterial activity against *H. pylori* than treatment with DD95 chitosans alone. The survival counts of three *H. pylori* strains at 50 μg/mL of amoxicillin, tetracycline, and metronidazole ranged 4.36 to 4.56 Log CFU/mL, 5.37 to 5.69 Log CFU/mL, and 4.69 to 5.11 Log CFU/mL, respectively. The survival counts gradually decreased with increasing concentrations (50 to 200 μg/mL) of antibiotics, with survival ranging from 2.25 to 4.28 Log CFU/mL at a concentration of 200 μg/mL of antibiotics. No bacterial counts were observed at 4000 μg/mL of tested antibiotics. Combination effects of antibiotics with 4000 μg/mL DD95 chitosan were observed. No survival was observed at a concentration of 50 μg/mL amoxicillin plus 4000 μg/mL DD95 chitosan or for the combination of 100 μg/mL tetracycline/metronidazole plus 4000 μg/mL DD95 chitosan. Overall, *H. pylori* counts in the presence of 50 μg/mL antibiotics of 3 tested antibiotics at pH 6.0 were decreased by 1.51–3.19 Log CFU/mL; while *H. pylori* counts were strongly decreased by 3.67–7.61 Log CFU/mL by adding 4000 μg/mL DD95 into the 50 μg/mL antibiotic-containing culture medium. 

As most antibiotics tend to be directed at tissues having a relatively neutral pH, some antibiotics may be unstable under acidic conditions. For example, amoxicillin may be degraded in simulated gastric fluid after three hours [24] and oral antibiotics for *H. pylori* infection may reduce the eradication rate in gastric juice having an acidic pH of 2.0. Indeed, in our study, the bacterial counts of *H. pylori* control strains cultured at pH 2.0 were lower (range 6.61 to 6.70 Log CFU/mL) (Table 4) than those cultured at pH 6.0 (range 7.20 to 7.59 Log CFU/mL) (Table 2). Lower survivals (range 3.97 to 4.52 Log CFU/mL) for 4000 μg/mL of DD95 chitosan at pH 2.0 than those at pH 6.0 (range 5.13 to 5.39 Log CFU/mL) (Table 3) were observed. The survival counts of three *H. pylori* strains cultured with 50 μg/mL of amoxicillin, tetracycline, and metronidazole at pH 2.0 ranged 3.83 to 4.08 Log CFU/mL, 4.18 to 5.23 Log CFU/mL, and 4.08 to 4.46 Log CFU/mL, respectively. Accordingly, *H. pylori* count was decreased by 1.47–2.82 Log CFU/mL in the 50 μg/mL antibiotics-added medium (pH 2.0), compared to those of control. Counts of *H. pylori* were greatly reduced by adding DD95 into antibiotic supplementary medium at pH 2.0. No bacterial counts were found in the combination of antibiotic and 4000 μg/mL DD95, irrespective of the antibiotic concentrations tested. *H. pylori* counts were strongly decreased by 6.61–6.70 Log CFU/mL at 4000 μg/mL DD95 plus 50 μg/mL antibiotics at pH 2.0. Thus, our results show that DD95 chitosan enhances the antibiotic drug activity against *H. pylori* strains.

Given the high bacterial load observed with *H. pylori* infection [25] and the gastric niche [26] where *H. pylori* could be protected from both antibiotic activity and an immune response, drug concentrations need to remain elevated for prolonged periods in the stomach, in order to achieve their therapeutic effectiveness [27]. The combination of antibiotics and DD95 chitosan may greatly reduce the dosage of antibiotics required for successful treatment of *H. pylori* infection. This approach is particularly useful for the treatment of drug resistant strains.

### 2.3. Adhesion of H. pylori to Human Gastric Carcinoma Cells (TSGH9201) in the Presence of DD95 Chitosan

**Figure 2:**
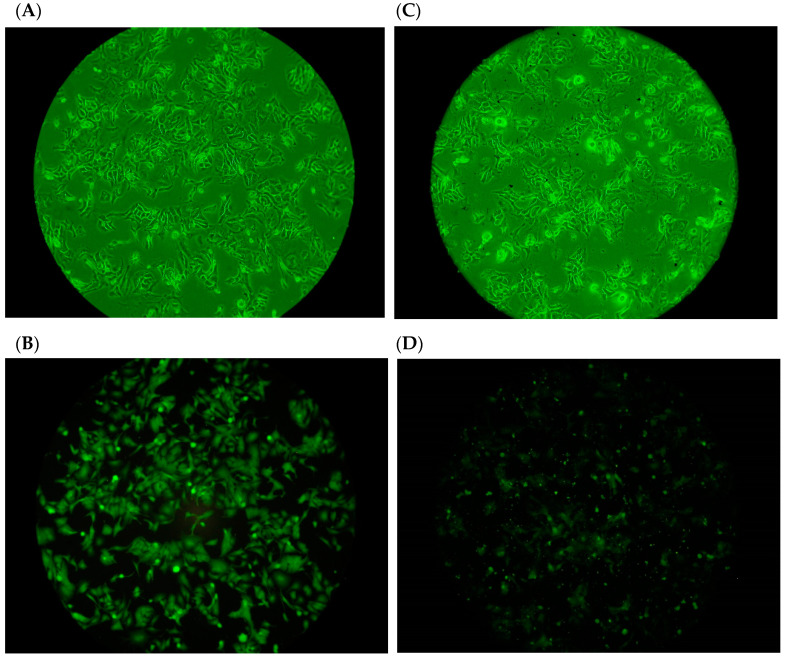
Effect of chitosan on the adhesion of *H. pylori* BCRC 17026 to TSGH cell line. (**A**) control, bright-field microscopic observation; (**B**) control, fluorescent microscopic observation; (**C**) chitosan (200 μg/mL) treatment, bright-field microscopic observation; (**D**) chitosan (200 μg/mL) treatment, fluorescent microscopic observation.

The effects of 200 μg/mL DD95 on the adhesion of the FITC-labeled *H. pylori* BCRC17026 to human gastric carcinoma cells are shown in Figure 2. For the control strains (not exposed to DD95), the FITC-labeled bacteria adhered to TSGH9201 cells and could be observed under both light (Figure 2A) and fluorescent microscopy (Figure 2C). The adhesion effects of bacteria were significantly reduced in the presence of DD95 treatment as observed using either light microscopy (Figure 2B) or fluorescent microscopy (Figure 2D). Explanations for the DD95-induced reduction in adhesion of *H. pylori* to TSGH9201 cells may include the reaction of DD95 with bacteria, which may retard bacterial binding with cells, or DD95 may react with cell surface residues, which prevent bacterial binding onto the cell surface. These potential activities merit further investigation to clarify the actual mechanism involved in DD95 interference with the adhesion of *H. pylori* to TSGH9201 cells.

Antibiotic therapy of *H. pylori* destroyed the microenvironment in the stomach leading to side effects and the rapid spread of resistant *H. pylori* strains [28]. Considering the long history chitosan use in a variety of food applications all over the world and the proposal that DD95 could represent a potential significant alternative antibacterial agent, this study using chitosan supporting its antagonistic activity toward *H. pylori* may lay the foundation for food applications able to contribute to the prevention of gastric diseases caused by *H. pylori* or as adjuvant therapy against *H. pylori* infections.

## 3. Material and Methods

### 3.1. Bacterial Strains, Cell Line, and Chemicals

*H. pylori* BCRC 15415, BCRC 10721, BCRC 10726, and TSGH 9201(human gastric carcinoma) were purchased from the Bioresources Collection and Research Center (Hsinchu, Taiwan). *H. pylori* No. 123, and 125 were obtained from Professor M.S.Wu, Division of Gastroenterology, National Taiwan University Hospital, Taipei, Taiwan. Acetic acid, acetonitrile, dimethyl sulfoxide (DMSO), glycerol, methanol, and sodium hydroxide (NaOH) were purchased from Fluka (Garage Gmbh, Buchs, Switzerland). Meanwhile, phenol red, amoxicillin, tetracycline, metronidazole, and sodium bicarbonate (NaHCO_3_) were purchased from Sigma Chemical Co. (Gillingham, UK). Chitin powder was obtained from Applied Chemical Co., Ltd. (Kaohsiung, Taiwan). Brain heart infusion broth (BHIB), tryptic soy broth (TSB), tryptic soy agar (TSA), bacto agar, urea broth and 50% egg yolk saline were supplied by Becton Dickinson (Sparks, MD, USA). RPMI-1640 medium and trypsin-EDTA were purchased from Gibco (New York, NY, USA)

### 3.2. DD95 Chitosan Preparation

Based on the method described by Chang, Lin, Wu and Tsai [16], a chitosan with 95.0% deacetylation degree, as measured using the colloid titration method [29], was obtained after deacetylation of a shrimp chitin powder suspension in 50% NaOH (1.0 g chitin per 13 mL NaOH) at 140 °C for 1 h.

### 3.3. Culture Conditions

*H. pylori* tested strains were stored in sBHIB (Brain heart infusion broth (BHIB) added with 10% horse serum) containing 50% sterile glycerol at –80 °C. To prepare the bacteria cultures, the strains stored at –80 °C were inoculated into sTSA (Tryptic soy agar (TSA) containing 5% horse serum) slant and incubated micro-aerobically (5% O_2_, 10% CO_2_, and 85% N_2_) by using anaerobic chamber with the micro-gas production pack (BBL Campypak 271034) at 37 °C for 72 h. All strains were sub-cultured twice in sBHIB and incubated micro-aerobically at 37 °C for 72 h. Finally, the cultures were diluted to 1.0 × 10^8^ CFU/mL with sterile 0.1% peptone water before use.

### 3.4. Antibacterial Test

The 1% chitosan stock solution was prepared by adding 0.2 g of DD95 to 10 mL distilled water, sterilizing at 121 °C for 15 min, and then adding 10 mL sterile 0.2 N HCl. In 50 mL-volume flasks containing 10 mL sBHIB, the 1% DD95 stock solution was added to obtain 1000, 2000, 3000, and 4000 μg/mL. Next, 1 mL of *H. pylori* culture were added to have an initial density of ca. 10^6^ CFU/mL, and incubated micro-aerobically at 37 °C for 6 h. Then, 0.1 mL aliquots of decimal dilutions of each culture were spread onto sTSA agar plates, and incubated micro-aerobically at 37 °C for 72 h before the colonies were counted. The experiments were run in triplicate. 

In another trial, the medium of sBHIB plus 4000 μg/mL was adjusted to pH 2.0, 3.0, 4.0, 5.0, 6.0, 7.0, and 8.0, respectively, using 0.1 N HCl and NaOH, before the *H. pylori* culture were added as described above to investigate the pH effects on the antibacterial activity of DD95.

### 3.5. Combination Effects of Antibiotic and DD95 Chitosan against H. pylori

The medium of sBHIB with/without 4000 μg/mL DD95 with pH value of pH 2.0 and pH 6.0 was added with the antibiotics of amoxicillin, tetracycline, and metronidazole to obtain the concentrations of 50, 100, 200, and 4000 μg/mL, respectively. Then, 1 mL of *H. pylori* culture were added to have an initial density of ca. 10^7^ CFU/mL, and incubated micro-aerobically at 37 °C for 6 h. The survival count was measured by spreading each sample on sTSA agar plates and incubated micro-aerobically at 37 °C for 72 h.

### 3.6. Urease Activity Assay

After treatment of *H. pylori* culture in sBHIB with/without DD 95 chitosan plus various amounts of antibiotics at 37 °C for 6 h, the urease production activity by *H. pylori* was evaluated, based on the method described by Adeniyi and Anyiam [18]. Briefly, the incubated *P. pylori* cultures were centrifuged (8800× *g*, 10 min, 4 °C). The collected sediments were washed twice with 0.02 M phosphate buffer solution (PBS, pH 6.8) and re-suspended in the same PBS (pH 6.8) for the urease activity assay. One milliter of the bacterial suspension was added to sterile test tube containing 0.03 M PBS (pH 6.8), 50 μL of 0.4% phenol red and 2% urea, mixed thoroughly and incubated at 37 °C for 30 min. The absorbance at 550 nm (A_550_) was measured using a colorimeter (UV-160-A, Shimadzu, Tokyo, Japan):

Relative urease activity (%) = (A_550 of Sample_/A_550 of Control_) × 100%.

### 3.7. Adherence of Fluorescein-Labeled H. pylori to Human Gastric Carcinoma Cells in the Presence of DD95 Chitosan

Human gastric carcinoma TSGH 9201 cells were cultured in RPMI1640 supplemented with 10% fetal calf serum (FCS) at 37 °C in an atmosphere of 5% CO_2_. Cells were sub-cultured every 2 days with the addition of fresh medium according to standard procedures. Based on the method of Tsai, et al. [30], TSGH 9201 cells (1 × 10^5^ cells/mL) on the well were added with 0.25% glutaraldehyde to prepare glutaraldehyde-fixed TSGH 9201 cells. Then, *H. pylori* cells collected from the sBHIB cultures were washed twice with PBS (pH 6.8) and re-suspended in 0.03M PBS (pH 6.8) to have a cell density of 10^6^ CFU/mL. This cell suspension was then mixed with equal volumes of fluorescein isothiocyanate (FITC) (2 mg/mL) at 20 °C for 30 min. After centrifugation (Himac CR-21, HITACHI, Tokyo, Japan) (8000× *g*, 20 min), bacteria were washed three times with PBS until no FITC residue in the supernatant. The FITC labeled *H. pylori* cells (1 × 10^6^ per well) with 200 μg/mL DD95 chitosan and without chitosan as control were added to glutaraldehyde-fixed TSGH 9201 cells, and cell plates were incubated at 37 °C with 5% CO_2_ for 1.5 h. After being washed three times with PBS, the cover slides were transferred to glass slides for a fluorescence microscopic observation.

### 3.8. Statistical Analysis

The data were analyzed using the general linear model (GLM) of Statistical Analysis System’s Procedures (Version 9.3, SAS Institute Inc., Cary, NC, USA) with a 5% level of significance. The means were separated using Duncan’s new multiple range test.

## 4. Conclusions

In this study, DD95 chitosan in medium clearly inhibited the growth and urease production by *H. pylori* and this inhibition effect was dose-dependent and pH dependent. The joint inhibitory effect of DD95 and the antibiotics of amoxicillin, tetracycline, and metronidazole on the growth and urease production of *H. pylori* strains were observed. This combination may represent a useful strategy in reducing the side effects observed with antibiotics therapy and shows strong potential as an alternative natural antibacterial agent. Moreover, DD95 chitosan significantly reduced the adherence of *H. pylori* onto human gastric carcinoma cells (TSGH 9201) indicating as a useful prevention agent from *H. pylori* infection.

## Figures and Tables

**Table 1:** The survival (%) ^1^ of *H. pylori* BCRC10726, No.123, No.125 in serum-BHIB ^2^ (pH 2.0–8.0) with/without chitosan (4000 μg/mL) and incubated micro-aerobically at 37 °C for 6 h.

pH	BCRC 10726			No.123			No.125		
	Bacterial Count ^2^ (log CFU/mL)		Survival (%)	Bacterial Count (log CFU/mL)		Survival (%)	Bacterial Count (log CFU/mL)		Survival (%)
	Control	Chitosan		Control	Chitosan		Control	Chitosan	
2	5.49 ± 0.01 ^D^	3.22 ± 0.06	0.53 ^D,3^	5.41 ± 0.02 ^D^	3.23 ± 0.03	0.65 ^D^	5.20 ± 0.03 ^E^	3.27 ± 0.03	1.17 ^D^
3	6.62 ± 0.01 ^C^	4.47 ± 0.01	0.70 ^D^	6.66 ± 0.05 ^C^	4.95 ± 0.06	1.94 ^D^	6.59 ± 0.02 ^D^	4.99 ± 0.05	3.51 ^D^
4	6.83 ± 0.03 ^B^	5.50 ± 0.00	4.71 ^C^	6.85 ± 0.05 ^B^	5.36 ± 0.05	3.22 ^D^	6.79 ± 0.05 ^C^	5.44 ± 0.03	4.51 ^D^
5	6.92 ± 0.03 ^B^	5.76 ± 0.01	6.93 ^B^	6.90 ± 0.06 ^B^	5.77 ± 0.03	7.46 ^C^	6.92 ± 0.04 ^B^	5.85 ± 0.03	8.62 ^C^
6	6.97 ± 0.02 ^A^	5.89 ± 0.00	8.29 ^B^	7.10 ± 0.07 ^A^	6.04 ± 0.08	8.74 ^C^	7.09 ± 0.02 ^A^	6.09 ± 0.03	10.02 ^C^
7	6.97 ± 0.01 ^A^	6.93 ± 0.02	89.90 ^A^	7.06 ± 0.06 ^A^	7.02 ± 0.06	90.72 ^B^	7.06 ± 0.02 ^A^	7.02 ± 0.04	89.89 ^B^
8	6.97 ± 0.00 ^A^	6.93 ± 0.01	91.63 ^A^	7.03 ± 0.01 ^A^	7.02 ± 0.01	96.63 ^A^	7.06 ± 0.02 ^A^	7.04 ± 0.03	94.30 ^A^

^1^ Survival (%) = (Count (CFU/mL) with chitosan/Count (CFU/mL) without chitosan) × 100%. ^2^ Values are mean ± SD of determination on three individual experiments. ^3^ Values in the same column with different capital letters (^A–E^) are significantly different ( *p* < 0.05).

**Table 2:** The relative urease activity ^1^ (%) of *H. pylori* BCRC10726, No.123, No.125 in serum-BHIB (pH 2.0–8.0) with/without chitosan (4000 μg/mL) and incubated micro-aerobically at 37 °C for 6 h.

pH	BCRC 10726			No. 123			No. 125		
	OD_550_		Relative Urease Activity (%)	OD_550_		Relative Urease Activity (%)	OD_550_		Relative Urease Activity (%)
	Control	Chitosan		Control	Chitosan		Control	Chitosan	
2	0.40 ± 0.00 ^C^	0.15 ± 0.00 ^2^	37.84 ± 0.35 ^D,3^	0.39 ± 0.01 ^C^	0.18 ± 0.00	45.58 ± 1.52 ^F^	0.41 ± 0.00 ^C^	0.19 ± 0.01	46.58 ± 0.37 ^F^
3	0.41 ± 0.00 ^C^	0.18 ± 0.01	43.72 ± 0.35 ^C^	0.40 ± 0.01 ^C^	0.19 ± 0.01	48.63 ± 1.93 ^E^	0.42 ± 0.00 ^C^	0.22 ± 0.00	51.93 ± 0.64 ^E^
4	0.44 ± 0.01 ^A^	0.21 ± 0.00	47.82 ± 0.98 ^B^	0.44 ± 0.01 ^A^	0.22 ± 0.00	49.25 ± 0.58 ^D,E^	0.45 ± 0.00 ^A^	0.25 ± 0.01	54.84 ± 0.54 ^D^
5	0.42 ± 0.00 ^B^	0.20 ± 0.01	48.61 ± 1.08 ^B^	0.43 ± 0.01 ^A,B^	0.22 ± 0.00	50.51 ± 0.69 ^C,D^	0.44 ± 0.00 ^B^	0.24 ± 0.00	56.02 ± 0.41 ^C,D^
6	0.41 ± 0.00 ^C^	0.20 ± 0.01	49.51 ± 0.25 ^B^	0.42 ± 0.00 ^B^	0.22 ± 0.01	51.34 ± 0.51 ^C^	0.43 ± 0.01 ^B^	0.25 ± 0.00	47.39 ± 1.09 ^C^
7	0.20 ± 0.00 ^D^	0.20 ± 0.00	97.54 ± 0.50 ^A^	0.21 ± 0.01 ^D^	0.20 ± 0.01	93.45 ± 1.06 ^B^	0.21 ± 0.01 ^D^	0.20 ± 0.01	94.85 ± 0.61 ^B^
8	0.20 ± 0.01 ^D^	0.20 ± 0.01	97.35 ± 0.71 ^A^	0.21 ± 0.01 ^D^	0.20 ± 0.01	97.89 ± 0.24 ^A^	0.21 ± 0.01 ^D^	0.20 ± 0.01	97.89 ± 0.63 ^A^

^1^ Relative urease activity (%) = (OD_550 of Sample_ /OD_550 of Control_) × 100%. ^2^ Values are mean ± SD of determination on three individual experiments. ^3^ Values in the same column with different capital letters (^A–F^) are significantly different (*p* < 0.05).

**Table 3:** Effect of various concentrations of antibiotics with/without chitosan against of *H. pylori* BCRC 10726, No. 123, No. 125 at pH 6.0 for 6 h.

Antibiotic (μg/mL)		BCRC 10726		No. 123		No. 125	
		Bacterial Count (Log CFU/mL)		Bacterial Count (Log CFU/mL)		Bacterial Count (Log CFU/mL)	
		Chitosan conc. (μg/mL)		Chitosan conc. (μg/mL)		Chitosan conc. (μg/mL)	
		0	4000	0	4000	0	4000
**Control**		7.20 ± 0.03 ^1^	5.32 ± 0.10	7.61 ± 0.02	5.13 ±0.05	7.59 ± 0.01	5.39 ± 0.07
Amoxicillin	50	4.36 ± 0.01	0.00 ± 0.00	4.42 ± 0.01	0.00 ± 0.00	4.56 ± 0.01	0.00 ± 0.00
	100	3.26 ± 0.01	0.00 ± 0.00	4.31 ± 0.01	0.00 ± 0.00	4.33 ± 0.01	0.00 ± 0.00
	200	2.25 ± 0.05	0.00 ± 0.00	3.74 ± 0.04	0.00 ± 0.00	4.04 ± 0.02	0.00 ± 0.00
	4000	0.00 ± 0.00	-	0.00 ± 0.00	-	0.00 ± 0.00	-
Tetracycline	50	5.69 ± 0.02	3.24 ± 0.02	5.37 ± 0.05	3.84 ± 0.03	5.39 ± 0.01	3.92 ± 0.02
	100	5.02 ± 0.03	0.00 ± 0.00	4.75 ± 0.01	0.00 ± 0.00	4.78 ± 0.01	0.00 ± 0.00
	200	3.98 ± 0.02	0.00 ± 0.00	2.80 ± 0.04	0.00 ± 0.00	3.22 ± 0.01	0.00 ± 0.00
	4000	0.00 ± 0.00	-	0.00 ± 0.00	-	0.00 ± 0.00	-
Metronidazole	50	4.69 ± 0.01	2.46 ± 0.02	5.07 ± 0.01	3.38 ± 0.02	5.11 ± 0.02	3.64 ± 0.02
	100	4.55 ± 0.02	0.00 ± 0.00	3.89 ± 0.00	0.00 ± 0.00	3.95 ± 0.00	0.00 ± 0.00
	200	4.28 ± 0.02	0.00 ± 0.00	3.10 ± 0.02	0.00 ± 0.00	3.34 ± 0.02	0.00 ± 0.00
	4000	0.00 ± 0.00	-	0.00 ± 0.00	-	0.00 ± 0.00	-

^1^ Values are mean ± SD of determination on three individual experiments. -: Not detected.

**Table 4:** Effect of various concentrations of antibiotics with/without chitosan against of *H. pylori* BCRC 10726, No. 123, No. 125 at pH 2.0 for 6 h.

Antibiotic (μg/mL)		BCRC 10726		No. 123		No. 125	
		Bacterial Count (Log CFU/mL)		Bacterial Count (Log CFU/mL)		Bacterial Count (Log CFU/mL)	
		Chitosan (μg/mL)		Chitosan (μg/mL)		Chitosan (μg/mL)	
		0	4000	0	4000	0	4000
**Control**		6.70 ± 0.03 ^1^	3.97 ± 0.06	6.61 ± 0.02	4.28 ± 0.02	6.66 ± 0.04	4.52 ± 0.01
Amoxicillin	50	4.08 ± 0.03	0.00 ± 0.00	3.83 ± 0.01	0.00 ± 0.00	3.84 ± 0.00	0.00 ± 0.00
	100	3.44 ± 0.02	0.00 ± 0.00	3.60 ± 0.01	0.00 ± 0.00	3.63 ± 0.01	0.00 ± 0.00
	200	3.08 ± 0.03	0.00 ± 0.00	3.30 ± 0.01	0.00 ± 0.00	3.40 ± 0.00	0.00 ± 0.00
	4000	0.00 ± 0.00	-	0.00 ± 0.00	-	0.00 ± 0.00	-
Tetracycline	50	5.23 ± 0.03	0.00 ± 0.00	4.39 ± 0.02	0.00 ± 0.00	4.18 ± 0.02	0.00 ± 0.00
	100	4.58 ± 0.01	0.00 ± 0.00	3.75 ± 0.01	0.00 ± 0.00	3.02 ± 0.02	0.00 ± 0.00
	200	3.39 ± 0.01	0.00 ± 0.00	1.79 ± 0.01	0.00 ± 0.00	2.47 ± 0.02	0.00 ± 0.00
	4000	0.00 ± 0.00	-	0.00 ± 0.00	-	0.00 ± 0.00	-
Metronidazole	50	4.23 ± 0.00	0.00 ± 0.00	4.08 ± 0.01	0.00 ± 0.00	4.46 ± 0.00	0.00 ± 0.00
	100	4.10 ± 0.05	0.00 ± 0.00	2.91 ± 0.00	0.00 ± 0.00	3.87 ± 0.03	0.00 ± 0.00
	200	3.84 ± 0.02	0.00 ± 0.00	2.10 ± 0.02	0.00 ± 0.00	2.49 ± 0.02	0.00 ± 0.00
	4000	0.00 ± 0.00	-	0.00 ± 0.00	-	0.00 ± 0.00	-

^1^ Values are mean ± SD of determination on three individual experiments. -: Not detected.

## References

[1] Moss S.F. (2017). The clinical evidence linking Helicobacter pylori to gastric cancer. Cell. Mol. Gastroenterol. Hepatol..

[2] Zamani M., Ebrahimtabar F., Zamani V., Miller W., Alizadeh-Navaei R., Shokri-Shirvani J., Derakhshan M. (2018). Systematic review with meta-analysis: The worldwide prevalence of Helicobacter pylori infection. Aliment. Pharmacol. Ther..

[3] Thung I., Aramin H., Vavinskaya V., Gupta S., Park J., Crowe S., Valasek M. (2016). the global emergence of Helicobacter pylori antibiotic resistance. Aliment. Pharmacol. Ther..

[4] Papastergiou V., Georgopoulos S.D., Karatapanis S. (2014). Treatment of Helicobacter pylori infection: Meeting the challenge of antimicrobial resistance. World J. Gastroenterol. WJG.

[5] Harb A.H., El Reda Z.D., Sarkis F.S., Chaar H.F., Sharara A.I. (2015). Efficacy of Reduced-Dose Regimen of a Capsule Containing Bismuth Subcitrate, Metronidazole, and Tetracycline Given with Amoxicillin and Esomeprazole in the Treatment of Helicobacter Pylori Infection.

[6] Perri F., Festa V., Merla A., Quitadamo M., Clemente R., Andriulli A. (2002). Amoxicillin-Tetracycline Combinations are Inadequate as Alternative Therapies for Helicobacter pylori Infection. Helicobacter.

[7] Huang Y.-Q., Huang G.-R., Wu M.-H., Tang H.-Y., Huang Z.-S., Zhou X.-H., Yu W.-Q., Su J.-W., Mo X.-Q., Chen B.-P. (2015). Inhibitory effects of emodin, baicalin, schizandrin and berberine on hefA gene: Treatment of Helicobacter pylori-induced multidrug resistance. World J. Gastroenterol. WJG.

[8] Cardoso O., Donato M.M., Luxo C., Almeida N., Liberal J., Figueirinha A., Batista M.T. (2018). Anti-Helicobacter pylori potential of Agrimonia eupatoria L. and Fragaria vesca. J. Funct. Foods.

[9] Sharifi A., Azizi M., Moradi-Choghakabodi P., Aghaei S., Azizi A. (2019). In vitro anti-Helicobacter pylori activity of aqueous extract from Persian Oak testa. Chin. Herb. Med..

[10] Spósito L., Oda F.B., Vieira J.H., Carvalho F.A., dos Santos Ramos M.A., de Castro R.C., Crevelin E.J., Crotti A.E.M., Santos A.G., da Silva P.B. (2019). In vitro and in vivo anti-Helicobacter pylori activity of Casearia sylvestris leaf derivatives. J. Ethnopharmacol..

[11] Mendes J., Paschoalin R., Carmona V., Neto A.R.S., Marques A., Marconcini J., Mattoso L., Medeiros E., Oliveira J. (2016). Biodegradable polymer blends based on corn starch and thermoplastic chitosan processed by extrusion. Carbohydr. Polym..

[12] Notario-Pérez F., Martín-Illana A., Cazorla-Luna R., Ruiz-Caro R., Bedoya L.-M., Tamayo A., Rubio J., Veiga M.-D. (2017). Influence of chitosan swelling behaviour on controlled release of tenofovir from mucoadhesive vaginal systems for prevention of sexual transmission of HIV. Mar. Drugs.

[13] Hu Z., Zhang D.-Y., Lu S.-T., Li P.-W., Li S.-D. (2018). Chitosan-based composite materials for prospective hemostatic applications. Mar. Drugs.

[14] Gong Y., Tao L., Wang F., Liu W., Jing L., Liu D., Hu S., Xie Y., Zhou N. (2015). Chitosan as an adjuvant for a Helicobacter pylori therapeutic vaccine. Mol. Med. Rep..

[15] Verlee A., Mincke S., Stevens C.V. (2017). Recent developments in antibacterial and antifungal chitosan and its derivatives. Carbohydr. Polym..

[16] Chang S.-H., Lin H.-T.V., Wu G.-J., Tsai G.J. (2015). pH Effects on solubility, zeta potential, and correlation between antibacterial activity and molecular weight of chitosan. Carbohydr. Polym..

[17] Singh D.Y., Prasad N.K. (2011). Double liposomes mediated dual drug targeting for treatment of Helicobacter pylori infections. Die Pharm.-Int. J. Pharm. Sci..

[18] Adeniyi B.A., Anyiam F.M. (2004). In vitro anti-Helicobacter pylori potential of methanol extract of Allium ascalonicum Linn. (Liliaceae) leaf: Susceptibility and effect on urease activity. Phytother. Res. Int. J. Devoted Pharmacol. Toxicol. Eval. Nat. Prod. Deriv..

[19] Chantarasataporn P., Tepkasikul P., Kingcha Y., Yoksan R., Pichyangkura R., Visessanguan W., Chirachanchai S. (2014). Water-based oligochitosan and nanowhisker chitosan as potential food preservatives for shelf-life extension of minced pork. Food Chem..

[20] Tsai G.J., Su W.H., Chen H.C., Pan C.L. (2002). Antimicrobial activity of shrimp chitin and chitosan from different treatments and application to fish preservation. Fish. Sci..

[21] Chang S.H., Chen C.H., Tsai G.J. (2020). Effects of chitosan on *Clostridium perfringens* and application in the preservation of pork sausage. Mar. Drugs.

[22] Hoffman J. (1997). Pharmacological therapy of Helicobacter pylori infection. Semin. Gastrointest. Dis..

[23] Chiba N. (2019). Ulcer disease and Helicobacter pylori infection: Current treatment. Evid.-Based Gastroenterol. Hepatol..

[24] Ramteke S., Ganesh N., Bhattacharya S., Jain N.K. (2009). Amoxicillin, clarithromycin, and omeprazole based targeted nanoparticles for the treatment of H. pylori. J. Drug Target..

[25] Molnar B., Szoke D., Ruzsovics A., Tulassay Z. (2008). Significantly elevated Helicobacter pylori density and different genotype distribution in erosions as compared with normal gastric biopsy specimen detected by quantitative real-time PCR. Eur. J. Gastroenterol. Hepatol..

[26] Ricci V., Zarrilli R., Romano M. (2002). Voyage of Helicobacter pylori in human stomach: Odyssey of a bacterium. Dig. Liver Dis..

[27] Wu T., Wang L., Gong M., Lin Y., Xu Y., Ye L., Yu X., Liu J., Liu J., He S. (2019). Synergistic effects of nanoparticle heating and amoxicillin on H. pylori inhibition. J. Magn. Magn. Mater..

[28] Chen X., Liu X.M., Tian F., Zhang Q., Zhang H.P., Zhang H., Chen W. (2012). Antagonistic activities of lactobacilli against Helicobacter pylori growth and infection in human gastric epithelial cells. J. Food Sci..

[29] Toei K., Kohara T. (1976). A conductomeric method for colloid titrations. Anal. Chim. Acta.

[30] Tsai C.-C., Huang L.-F., Lin C.-C., Tsen H.-Y. (2004). Antagonistic activity against Helicobacter pylori infection in vitro by a strain of Enterococcus faecium TM39. Int. J. Food Microbiol..

